# Optimal Artificial Neural Network Type Selection Method for Usage in Smart House Systems

**DOI:** 10.3390/s21010047

**Published:** 2020-12-24

**Authors:** Vasyl Teslyuk, Artem Kazarian, Natalia Kryvinska, Ivan Tsmots

**Affiliations:** 1 Department of Automated Control Systems, Lviv Polytechnic National University, 79013 Lviv, Ukraine; artem.h.kazarian@lpnu.ua (A.K.); ivan.h.tsmots@lpnu.ua (I.T.); 2 Department of Information Systems, Faculty of Management, Comenius University in Bratislava, Bratislava 25 82005, Slovakia; natalia.kryvinska@uniba.sk

**Keywords:** smart house, artificial neural network (ANN) algorithm, feedforward neural network, recurrent neural network, long short-term memory, gated recurrent unit

## Abstract

In the process of the “smart” house systems work, there is a need to process fuzzy input data. The models based on the artificial neural networks are used to process fuzzy input data from the sensors. However, each artificial neural network has a certain advantage and, with a different accuracy, allows one to process different types of data and generate control signals. To solve this problem, a method of choosing the optimal type of artificial neural network has been proposed. It is based on solving an optimization problem, where the optimization criterion is an error of a certain type of artificial neural network determined to control the corresponding subsystem of a “smart” house. In the process of learning different types of artificial neural networks, the same historical input data are used. The research presents the dependencies between the types of neural networks, the number of inner layers of the artificial neural network, the number of neurons on each inner layer, the error of the settings parameters calculation of the relative expected results.

## 1. Introduction

Today one of the main functions of “smart” home systems is to collect the data from sensors connected to the system, which are located in the houses and apartments of users. The generated data stream from sensors can be used to process them in real time, as well as when processing large amounts of data to predict and model potential situations that will require certain actions from the system functionality logic, which will help to change the system settings in advance for rapid automated system responses to emerging situations. During the work of such systems in residential buildings, such situations may include: a change in the number of users present in the premises; a change of climatic conditions outside the house; a change of day time; a power outage; and a sequence of regular “smart” house system user actions, which requires the automation of changes in the settings of household appliances using algorithms laid down during the system’s development.

In scope of complex solution synthesis that focuses on automating the settings controls for various appliances in the house, the main problem is related to choosing the optimal type of artificial neural network, which will provide the best results of calculations for optimal temperature level, lighting and security in the house.

To solve this problem, we need:

- To develop the mathematical software based on the solution of the optimization problem, wherein the criterion for optimization should be the error of the ANN type;
- To develop research software and implement research directly.

The main target of this research is to develop the method for the simplification of artificial neural network choice, including such parameters as the best type of the ANN and the structure of the ANN’s hidden layers for better solution of the scientific problems with the help of a machine learning algorithm. Usage of this method will help to save time in the initial stage of research and to focus more on the data dependencies specifics, because the machine learning part will be configured automatically, according to the type of historical data used in the scope of the research.

The structure of the article includes the following: an introduction, which indicates the relevance of the study and a review of the literature on the subject of the work; in the first section the optimization problem is formulated and the input data for the research are defined; the second section presents the development process of the method for selecting the optimal ANN type; the third section describes the method for determining the optimal number of hidden layers and neurons in ANN; the fourth section describes the features of software implementation, and the fifth presents the results and analyses.

## 2. Related Works

Today, “smart” house systems have occupied one of the key niches in development in the field of intelligent information technology [1,2,3,4,5,6]. Such systems continuously generate a stream of data, that is used to solve a number of important tasks, namely, the automation of home appliances control [7,8], providing comfortable living conditions [9,10], reducing energy consumption [11,12], etc. Similar systems can also be used to monitor the elderly, children and pets [13,14,15]. The scope of “smart” house systems can be expanded by their usage for commercial purposes through their integration into the infrastructure of different functional-purpose premises (offices, shops, warehouses, government agencies, shopping and entertainment complexes, etc.) [16,17,18].

There are scientific papers [19,20,21] that describe the work of “smart” house systems using machine learning technologies, as well as some practical implementations of this idea among such developments as Nest Thermostat [22]. This product uses the machine learning algorithms to adapt settings to user preferences and seasons. Another product by Netatmo [23] makes it possible to identify the presence of certain users in the room, using face recognition technologies to warn when an unidentified person appears in the room. Amazon Echo [24] and Google Home [25] allow you to control third-party devices using voice commands. Klug Home uses machine learning algorithms to set the temperature levels of the air conditioner, which are preferred by users, taking into account the time of day and the activity of people in the room, as well as to detect abnormal increases in electricity consumption [26]. The peculiarity of the available products is that they interact with a limited number of home appliances, so the development of a comprehensive solution for managing a “smart” house system using machine learning algorithms is a topical issue of today. For solving problems of this type, the artificial neural network algorithms [27] are often used, which, in their turn, are divided into the several types used in various areas of human life automation.

## 3. Mathematical Formalization of the Optimization Problem

One of the most common tasks solved by “smart” house systems is the automated change of household appliances’ settings, according to the internal system logic, to ensure comfortable living conditions for the people in the house. This article formulates the task of “smart” house system development, using algorithms based on the ANN, which allows one to control appliances and configure home security system.

The main goal is to get the smallest value of error ($\Delta x_{\min}$) among the values of the device’s settings parameters in the house, calculated using a machine learning algorithm ($x_{\begin{array}{l} \mathrm{aut} \end{array}}$) and the parameters expected by the people inside the house ($x_{\exp}$). To solve this problem, several types of ANN are used (n), with the analysis and comparison of the desirable and actually received results for each type of neural network, in the automated management processes related to modes for thermostats, lighting devices and alarm systems, separately for each of the tasks:

(1) $$\Delta x_{\min}=\sum_{i=1}^{n}\left|x_{aut,i}-x_{\exp,i}\right|$$

where l≤i≤n.

In the process of research, a set of historical data was selected from 7510 records with parameters from the sensors and devices of a “smart” house. The data were obtained from a set collected over one year, from 200 sensors in Mitsubishi Electric Research Labs, and which were made public data [28]. To conduct the learning process of different types of neural networks, an identical training sample was used. This approach eliminates the influence of the specifics of the data used in the training process on the results of the ANN algorithm. The training sample contains information about thermostat settings (°C) in two rooms, lighting conditions (on/off) in each of the two rooms separately, security system settings (on/off alarm) for the whole house, and information about the presence of people in the house (indicators of motion sensors) in each room separately, as well as saving the data of the setting (ms. in Linux format) moment’s timestamp. An example of the data format in the training sample is shown in Table 1.

To ensure an optimal temperature setting, for the input data of the training sample the following parameters were used: timestamp; presence of people in room 1; presence of people in room 2; lighting in room 1; lighting in room 2; alarm mode. To solve the problem, the initial parameters that were expected to be obtained as a result of the artificial neural network algorithm were temperature in room 1 and temperature in room 2. The data format in the training sample is given in Table 2.

To ensure an optimal lighting mode, for the input data of the training sample the following parameters were used: timestamp; presence of people in room 1; presence of people in room 2; temperature regime in room 1; temperature in room 2; alarm mode. To solve the problem, the initial parameters that were expected to be obtained as a result of the artificial neural network algorithm were lighting in room 1 and lighting in room 2. An example of the data format in the training sample is shown in Table 3.

To ensure an optimal configuration of the home security system, the input data of the training sample included the following parameters: timestamp; presence of people in room 1; presence of people in room 2; temperature regime in room 1; temperature in room 2; lighting in room 1; lighting in room 2. To solve the problem, the initial parameter that weas expected to be obtained as a result of the artificial neural network algorithm was alarm mode. The data format in the training sample is given in Table 4.

**Figure 1:**
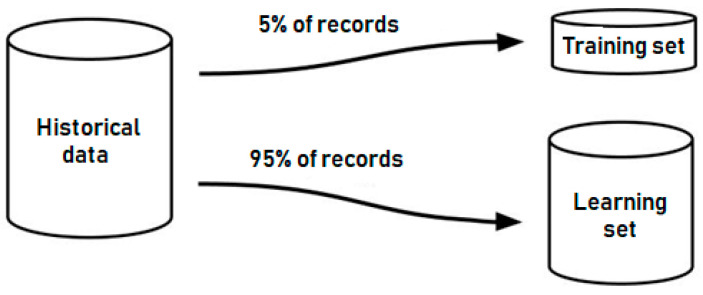
A scheme of historical data distribution between the test and educational datasets.

From the 7510 records of the historical data set, five percent of the records were deleted from the set and did not participate in the training sample, but were used in the process of testing the results of the ANN learning process (Figure 1).

The learning samples were formed to study the different types of ANN algorithms which will be used in the process of “smart” house household appliances control, and will also be used in the installed thermostat to ensure an optimal temperature, lighting and home security system mode.

## 4. The Method of an Optimal ANN Type Selection

The effectiveness of ANN directly depends on its type, its internal structure and the specifics of the problem. For example, to solve the image recognition problems in images and video streams, the most effective usage is convolutional neural networks [29], and for text translation, or programming bots that conduct dialogues with humans, the best choice is recurrent neural networks [30]. Therefore, there is a question of choosing the optimal artificial neural network structure, which will be used to automate the control of the temperature, security system and lighting in a “smart” house. The initial stage of choosing the artificial neural network structure is to create a list of known types of ANN [31,32,33,34,35]. Such a list in this example includes:

- Feedforward neural network [36];
- Recurrent neural network [37];
- Long short-term memory [38];
- Gated recurrent unit [39].

The next step of the method is to generate a training sample that will be used to study the ANN of the considered types. The training sample was generated by discarding 5% of the records that formed the test sample. This sample will be used in the future to calculate the error in the ANN algorithm’s results in comparison with the expected data. Such a percentage value of the training sample is chosen, similar to the traditional division of historical data sets used in the scope of machine learning algorithms’ training processes. This value is the approximate value between the amount of data that are used for efficient training and the amount of redundant data that cause memorization of the results by ANN.

The input parameters that will be fed into the input of the ANN ($x_{1},x_{2},\ldots x_{n}$) and the resulting parameters that the system must calculate according to the input data ($y_{1},y_{2},\ldots y_{p}$) were taken from a set of historical data collected from a temperature sensor, motion sensors, and other devices in the house. The next stage of the method is the sequential training and the usage of each artificial neural network type, and the analysis of the results. Having chosen the next type of ANN, the learning process was carried out with the help of the created training sample. Later, a trained artificial neural network was launched with the input of the test sample parameters, saving the obtained results. The error value was calculated for each output parameter (ΔY) by comparison of the obtained value of the corresponding parameter with the test sample:

(2) $$\Delta Y_{i}=\frac{Y}{Y_{test_{i}}}$$

Among the obtained values of errors for each output parameter ($\Delta Y_{i}$), the average value was calculated ($\Delta Y_{sum}$), which is the general error value for a particular type of ANN:

(3) $$\Delta Y_{sum}=\frac{\sum \Delta Y_{i}}{5}$$

After calculating the average error value, it was compared with the lowest obtained average error value of the previously considered types of artificial neural networks:

(4) $$\Delta Y_{sum}<\Delta Y_{\min}$$

If this value were to be less than the previous one, the current artificial neural network would be stored as the most optimal for solving the problem, and the average value of the error would be stored as the most optimal at a particular time-point in the research:

(5) $$\Delta Y_{\min}=\Delta Y_{sum}$$

The process of the training, start-up and analysis of the artificial neural network’s results was carried out sequentially for each type of ANN from the list, until all types were analyzed.

**Figure 2:**
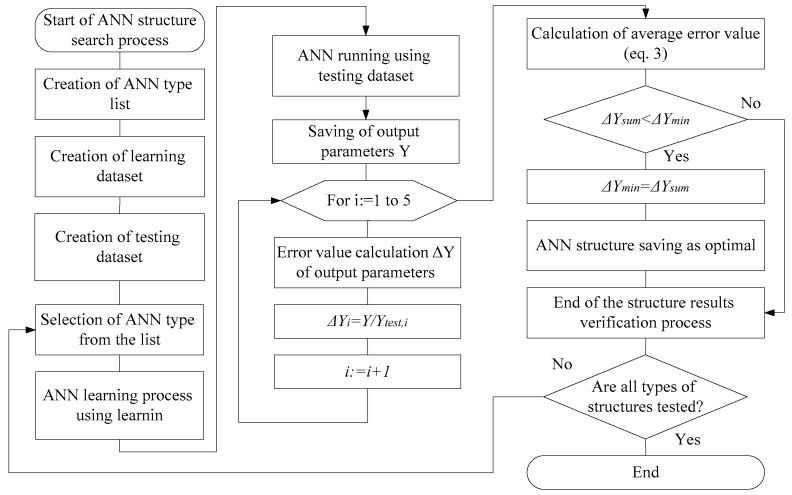
An algorithm of the method for the selection of the optimal type of neural network.

In this research, we present a method for finding an optimal type of artificial neural network, using appropriate training and test samples. The method is consistently used to solve problems in the automated control of temperature, lighting and security settings. The advantage of the developed method is the usage of a single set of historical data for the formation of training and test samples, by combining input and output parameters for each task. Such an approach eliminates the impact of the data specifics used in training on the ANN algorithm’s results. The developed algorithm for finding the optimal type of artificial neural network is shown in Figure 2.

## 5. The Selection Method of ANN Layers and Neuron Number

An important task in determing the efficient artificial neural network algorithm to be used is to develop a method for selecting the optimal artificial neural network with a hidden layers structure, and sprecifically, the number of inner layers (Nh) and the number of neurons in each layer (N[l]).

The developed algorithm for finding the optimal structure of the ANN’s hidden layers includes the next steps.

**Step 1.** Determine the variables of the ANN’s hidden layers number (L), the number of neurons on each layer (N_L_), and their initial values:

L = 1, N_L_ = 1 (6)

**Step 2.** Perform the training of the artificial neural network with the internal structure of neurons, which corresponds to the current value of the artificial neural network’s hidden layers number L, and the number of neurons on each layer N_L_. The training process has strict constraints that allow one to conduct efficient ANN training without the redundant usage of hardware computation resources. The network will stop training whenever one of the following two criteria is met: the training error has gone below the threshold (0.005), or the max number of iterations (20,000) has been reached.

**Step 3.** Check the results of the ANN’s output.

Run the ANN algorithm with a test sample, with the input containing the values of real historical data ($Y_{r}$), and the resulting parameters calculated by the algorithm are obtained ($Y_{calc}$). The calculation of an error in percents (ΔY) for each output parameter (values of energy consumption and operating time) proceeds as follows:

(7) $$\Delta Y_{i}=\frac{\left|Y_{calc}{}_{i}-Y_{ri}\right|}{Y_{ri}}$$

where i is a sequence number of the parameter, $Y_{ri}$ is the value of the parameter from the test sample, and $Y_{calci}$ is the value of the output parameter calculated by the algorithm.

The value of the error of the artificial neural network algorithm with the current structure of hidden layers ($\Delta Y_{struct}$) is considered as an arithmetic value of the errors obtained for each individual parameter:

(8) $$\Delta Y_{struct}=\frac{\sum_{i=1}^{n}\Delta Y_{i}}{n}$$

where n is the number of output parameters, i is the sequence number of the parameter, and $\Delta Y_{i}$ is the percentage value of the error calculated for the current parameter.

**Step 4.** Implement the ranking of the created structures according to the efficiency of the work.

The created hidden layers structure of the artificial neural network is added to the list of previously created structures with the current number of hidden layers (L), sorted by the error values of the algorithm, starting from the smallest.

**Step 5.** Check the conditions for adding a new neuron to the current hidden layer.

If the value of the error of the created structure is not less than the value of the third in the list of the previously created structures during the last five iterations, go to the next step of the algorithm (Step 6). Otherwise, add an additional neuron to the current hidden layer (Li):

N_L_ = N_L_ + 1 (9)

where N_L_ is the number of neurons in the hidden layer L.

After adding the additional neuron to the last ANN layer, we return to Step 2 of the algorithm, that is the training of the ANN with the newly created structure of the hidden layers.

**Step 6.** Check the conditions of the completion of the algorithm.

The structure with the lowest error value on the list of structures with the current number of hidden layers (L) is added to the general list of the most optimum created structures, sorted by values of algorithm errors, beginning from the smallest. If the value of the structure error added to the list is not less than the value of the third on the list of previously added structures during the last five iterations, we complete the algorithm by finding an optimal internal structure of the ANN. The most efficient structure is considered to be the first structure in the list with the lowest arithmetic error value of all the automatically determined initial parameters of the artificial neural network. Otherwise, we move on to Step 7 of the algorithm to increase the number of the artificial neural network’s hidden layers.

**Step 7.** Determine the number of the ANN structure’s hidden layers for the next cycle.

Add an additional hidden layer with one neuron to the most effective structure of an artificial neural network created at the current moment:

L = L + 1, N_L_ = 1 (10)

**Figure 3:**
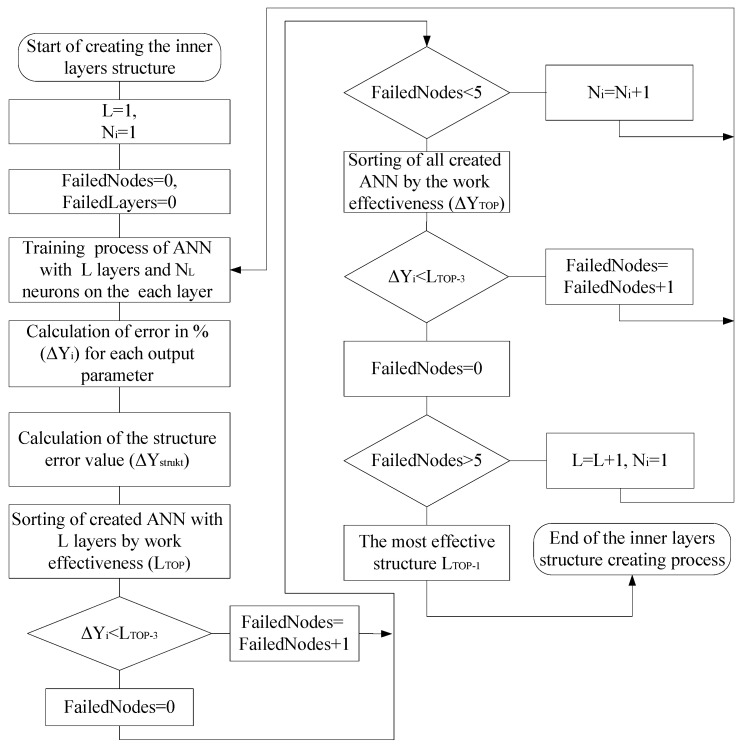
A block diagram of the algorithm for creating the ANN’s inner layers’ structure.

After adding the additional ANN hidden layer with one initial neuron, we return to Step 2 of the algorithm, that is, the training of the ANN with the newly created structure of the hidden layers. The described algorithm for the search for the optimal ANN hidden layers structure is shown in Figure 3.

## 6. Features of the Created Methods for Software Implementation

The software implementation of the optimal ANN type selection method is based on the usage of NodeJS technology [40], which has proven to be a fast technology for processing a large amount of simultaneously generated events. This specificity is common in “smart” house systems, the developers of which face the problem of optimizing data flows generated by sensors located in the “smart” house [41]. To implement the artificial neural network algorithm, the BrainJS library was used [42], which allows one to flexibly adjust the parameters and types of ANN and to train the artificial neural network algorithm with the help of a training dataset. To save the data of the training dataset, a non-relational database MongoDB was used [43].

Additionally, an algorithm has been developed to find an optimal type of ANN, which includes steps to learn, test and analyze the results of each type of artificial neural network. This algorithm allows one to use the most effective type of ANN for solving the problems of automated temperature control, lighting and home security. The developed software allows us to carry out the processes of training and testing, and to obtain the results of the ANN algorithms of each considered type for further analysis of the efficiency of their use.

The implemented ANN is based on the usage of the brain.js library, which is implemented in the JavaScript programming language and runs on the NodeJS platform.

The brain.js library allows one to configure the learning process parameters of the created ANN, such as the allowable error threshold for the training sample, the maximum number of learning iterations, and others. For the developed system, the allowable error threshold is 0.015 (1.5%), and the maximum number of learning iterations is 20,000.

## 7. The Results of Research and Usage Results Comparison for Different Types of Artificial Neural Networks

During the research, the results of different types of artificial networks have been compared.

### 7.1. The Results of the Feedforward Neural Network Research

**Figure 4:**
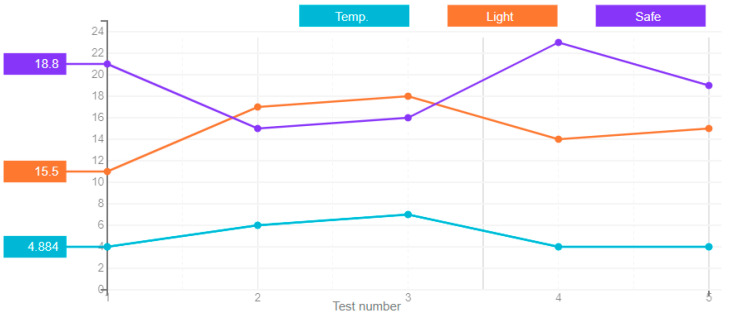
A chart of the feedforward neural network’s error value.

In a feedforward neural network, the signals propagate in one direction, starting from the input layer of neurons, through the hidden layers to the output layer, and on the output neurons the result of signal processing is obtained. There are no back connections in this type of network. The results of the artificial neural network error for this type, derived over five attempts, are shown in Figure 4.

### 7.2. The Results of the Recurrent Artificial Neural Network Research

**Figure 5:**
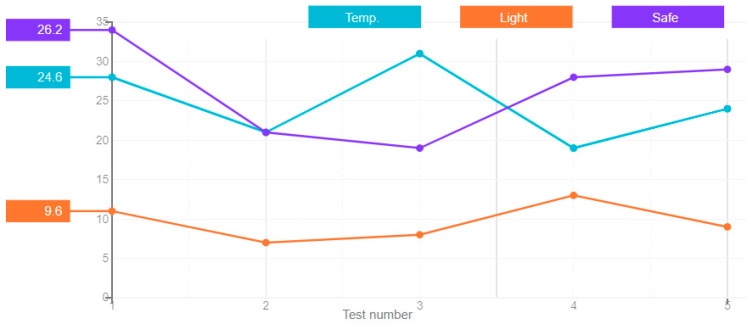
A chart of the recurrent neural network’s error values.

In the recurrent neural networks, connections between nodes form a time-oriented graph. This creates an internal state of the network that allows us to exhibit dynamic behavior over time. Unlike the feedforward neural networks, the recurrent neural networks can use their internal memory to process arbitrary input sequences. The obtained results of the error of this ANN type in five attempts are shown in Figure 5.

### 7.3. The Results of the Long Short-Term Memory Research

**Figure 6:**
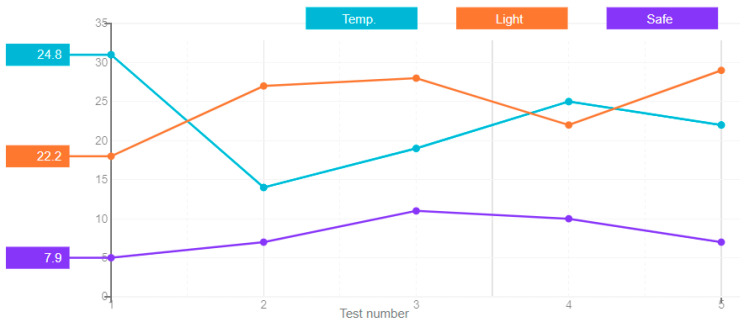
A graph of the long short-term memory ANN’s error value.

The long short-term memory is an architecture of the recurrent neural networks, which like most recurrent neural networks, is universal when there is a sufficient number of network nodes, which allows us to compute anything that a normal computer can compute, if it has an appropriate matrix of weights that can be considered as its program. Unlike traditional recurrent neural networks, the long short-term network is well suited for experiential learning, to classify, process, or predict time series when there are time delays of unknown duration between important events. The obtained results of the error of this artificial neural network type in five attempts are shown in Figure 6.

### 7.4. The Results of the Gated Recurrent Unit ANN Research

**Figure 7:**
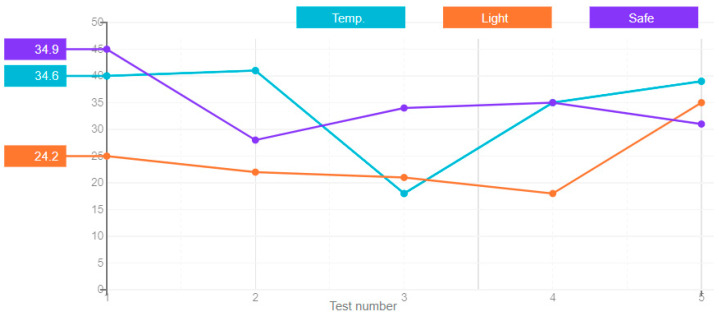
A chart of the gated recurrent units ANN’s error values.

The gated recurrent units are valve mechanisms in the recurrent neural networks that are similar to long short-term memory with a forget valve, but have fewer parameters because they do not have an output valve. The results of the error of the artificial neural network of this type in five attempts are shown in Figure 7.

### 7.5. The Results of the ANN Internal Structure Synthesis Peculiarities

**Figure 8:**
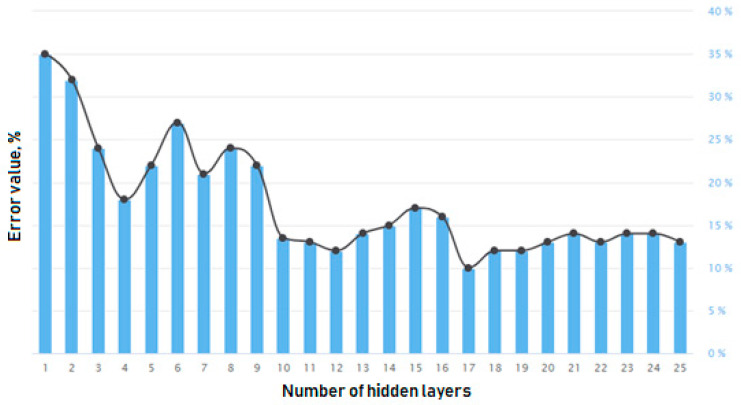
A chart of the ANN’s error value algorithm with the created structures.

The results of the algorithm (Figure 3) show that the error value of the artificial neural network’s hidden layers structure decreases rapidly in the initial algorithm steps (Figure 8), reaches its minimum in a structure with 17 hidden layers, and gradually turns the structure into a redundant one without noticeable improvement of the algorithm’s efficiency. Therefore, an important condition for the algorithm is strict compliance with the stop criteria, which are configured to determine the period of deterioration in the performance of the search process for an optimal structure of the artificial neural network’s hidden layers.

## 8. The Research Results Analysis

During the analysis of the obtained research results, in the process of comparing the obtained results of ANN algorithms with different types, we can conclude that to solve different problems with the help of the “smart” house system, it is advisable to use different types of artificial neural networks, depending on the specifics of the task. To adjust the temperature in the room, the best choice is to use the feedforward neural networks. This choice provides the error of the obtained results in the range of less than 5%. To configure the lighting fixtures of the apartment, with the installed system of the “smart” house, the smallest value of error between the obtained and desired results of the ANN algorithm has been obtained using the recurrent neural networks. This solution provides an error of less than 10%. To ensure the optimal mode of the security of an apartment equipped with a system of “smart” house, the best choice is the artificial neural network, i.e., long short-term memory, which provides an error between the obtained and desired results of less than 8%. As a result of comparing the obtained errors with the obtained real and desired settings of household appliances in the house, the usage of the gated recurrent unit of the recurrent neural networks is not a relevant solution, due to the big values of the errors.

Therefore, the results of using different types of artificial neural networks to solve problems have been analyzed, the error values between the obtained and expected results have been calculated, and the optimal types of the ANN for each problem have been selected separately.

In addition, the analysis of the selected ANN types’ results with the same basic structures of inner layers, which have been taken by default, show that in solving various tasks, such as temperature control and the selection of security system settings, the output results of the ANN have a large error value difference compared to the test dataset values. The analysis of the research results related to the dependence of the number of the ANN’s internal hidden layers, and the number of neurons in each hidden layer, on the output results of the error value of the automated decision-making mechanism shows that the usage of the developed algorithm allows us to find an optimal hidden ANN structure independent of the context of the problem we need to solve.

The analyzed results show that the developed method can improve the results of the machine learning algorithms used in the sphere of “smart” home systems development. In addition, such a method can be used in other spheres that have a similar workflow of problem-solving, with an available big amount of historical data that can be analyzed, such as medicine, retail, etc. The presented method can be improved by adding new ANN types that will take part in the stage of best ANN type selection. The main challenge of this research was related to the high level of hardware computational possibilities during the method’s implementation. The ANN training is a high-load process, and in terms of method logic, it is multiplicated on a few different ANNs that trained simultaneously to minimize the time required for getting results.

## 9. Conclusions

A method for the selection of an optimal type of ANN has been developed. It is based on the idea of the practical usage of several ANN types and the further calculation of the error values for each type using identical data sets for ANN training, which eliminates the influence of data specificity on the training sample. The software has been developed that allows us to conduct the learning and testing of, and the deriving of results from, the ANN algorithm, such as the values of the optimal temperature in the rooms of the house, the lighting settings and the modes of the home security system’s operation. From the analysis of the obtained results, it follows that it is impossible to create one universal ANN as a complex solution for running the tasks related to the automated control of temperature modes, lighting devices and security systems in the room equipped with the system of the “smart” house. To solve each of these tasks, we need to use a separate artificial neural network of the specific type that provides the best performance, according to the specifics of the problem. An important task in the development of “smart” house systems using multiple ANNs is to create a mechanism of interaction between the created neural networks, to ensure no conflicts in the settings of the household appliances, and the correct choice of neural network types for each function of the developed system that uses artificial learning algorithms. In addition, to improve the accuracy of the results, it is necessary to use the search algorithm for the selection of an optimal ANN internal structure, which also cannot be universal, to solve the tasks of “smart” houses, and this significantly depends on the type of task. The combination of two created algorithms in the process of automated decision-making mechanism development for “smart” house systems provides a reduction in the results’ error values, regardless of the tasks to be solved.

## 10. Prospects for Future Research

The artificial neural network’s results were influenced not only by the chosen type and its internal structure, but also by other parameters that were not described in this research. The future research of this theme will be focused on the influence of these parameters on the output results, such as the type of activation function, the network weight initialization scheme, the maximum number of training iterations, and the training error.

## Figures and Tables

**Table 1:** A fragment of the training sample data.

Timestamp	Move in the Room 1	Move in the Room 2	Room Light 1	Room Light 2	Room Temp. 1	Room Temp. 2	Security Mode
1567519042	TRUE	FALSE	TRUE	TRUE	21	18	FALSE
1567523065	TRUE	TRUE	FALSE	FALSE	20	18	FALSE
1567535088	FALSE	TRUE	TRUE	FALSE	20	20	FALSE
1567560523	TRUE	FALSE	FALSE	FALSE	22	21	TRUE
...	...	...	...	...	...	...	...

**Table 2:** A fragment of the data of the training sample to ensure an optimal temperature.

Input Data						Output Data	
Timestamp	Move in the Room 1	Move in the Room 2	Room Light 1	Room Light 2	Security Mode	Room Temp. 1	Room Temp. 2
1567519042	TRUE	FALSE	TRUE	TRUE	FALSE	21	18
1567523065	TRUE	TRUE	FALSE	FALSE	FALSE	20	18
1567535088	FALSE	TRUE	TRUE	FALSE	FALSE	20	20
1567560523	TRUE	FALSE	FALSE	FALSE	TRUE	22	21
...	...	...	...	...	...	...	...

**Table 3:** A fragment of the training sample data to ensure an optimal lighting.

Input Data						Output Data	
Timestamp	Move in the Room 1	Move in the Room 2	Room Temp. 1	Room Temp. 2	Security Mode	Room Light 1	Room Light 2
1567519042	TRUE	FALSE	21	18	FALSE	TRUE	FALSE
1567523065	TRUE	TRUE	20	18	FALSE	FALSE	FALSE
1567535088	FALSE	TRUE	20	20	FALSE	FALSE	FALSE
1567560523	TRUE	FALSE	22	21	TRUE	FALSE	TRUE
...	...	...	...	...	...	...	...

**Table 4:** A fragment of the data from the training sample to ensure an optimal configuration of the home security system.

Input Data						Output Data	
Timestamp	Move in the Room 1	Move in the Room 2	Room Light 1	Room Light 2	Room Temp. 1	Room Temp. 2	Security Mode
1567519042	TRUE	FALSE	TRUE	TRUE	21	18	FALSE
1567523065	TRUE	TRUE	FALSE	FALSE	20	18	FALSE
1567535088	FALSE	TRUE	TRUE	FALSE	20	20	FALSE
1567560523	TRUE	FALSE	FALSE	FALSE	22	21	TRUE
...	...	...	...	...	...	...	...

## Data Availability

No new data were created or analyzed in this study. Data sharing is not applicable to this article.
